# Factors associated with female students’ past year experience of sexual violence in South African public higher education settings: A cross-sectional study

**DOI:** 10.1371/journal.pone.0260886

**Published:** 2021-12-02

**Authors:** Mercilene T. Machisa, Esnat D. Chirwa, Pinky Mahlangu, Yandisa Sikweyiya, Ncediswa Nunze, Elizabeth Dartnall, Managa Pillay, Rachel Jewkes

**Affiliations:** 1 South African Medical Research Council Gender and Health Research Unit, Pretoria, South Africa; 2 School of Public Health, Faculty of Health Sciences, University of Witwatersrand, Johannesburg, South Africa; 3 Sexual Violence Research Initiative, Pretoria, South Africa; 4 Department of Basic Education, Pretoria, South Africa; Universidad Pública de Navarra, SPAIN

## Abstract

**Background:**

Intimate partner sexual violence and non-partner rape experiences are widely reported by female students in South African higher education institutions, as they are globally. However, limited research has focused on investigating vulnerability factors, which is vital for informing interventions.

**Objective:**

To describe the factors and inter-relationships associated with female students’ increased vulnerability to past year experience of partner sexual violence and non-partner rape in South African higher education settings.

**Methods:**

We interviewed 1293 female students, i.e., 519 students in six Technical Vocational Education and Training (TVET) college campuses and 774 students at three university campuses. Participants were volunteers aged 18–30. The measured vulnerability factors included childhood sexual abuse, other trauma, mental ill-health, risky sexual behaviours, food insecurity, partner violence, and controlling behaviours. We used bivariate analysis, logistic regression, and structural equation modelling methods.

**Results:**

Twenty percent of participants experienced past-year sexual violence (17% partner sexual violence and 7.5% non-partner rape). Childhood sexual abuse had direct effects on experiencing past year sexual violence and physical, emotional partner violence or controlling behaviours. Risky sexual behaviours mediated the relationships of childhood sexual abuse or harmful alcohol use and past-year sexual violence experience. Mental ill-health mediated the relationships between childhood sexual abuse, other traumatic exposures, food insecurity, physical, emotional partner violence or controlling behaviours, and past-year partner sexual violence or non-partner rape experience.

**Conclusions:**

Risky sexual behaviours, gender inequitable relationship dynamics, mental ill-health, and food insecurity are related and amenable vulnerability factors associated with female students’ sexual violence experiences. Therefore, addressing these through comprehensive campus interventions, which are implemented when students first enrol in higher education and are most vulnerable to sexual violence, is critical. Society-wide sexual violence prevention is also imperative.

## Introduction

Researchers in the Global North have extensively contributed to the existing evidence, which focuses on the prevalence and individual factors which increase vulnerability for sexual violence (SV) experiences among college students [1–3]. In contrast, the contributions of researchers from the Global South are emergent. "SV includes rape or other sexual acts, attempted or perpetrated by intimate partners or non-partners using coercion, harassment, physical force or other tactics such as psychological intimidation, emotional blackmail and threats of physical harm" [4]. While SV against female students is a widely acknowledged problem within South African higher education settings, quantitative data indicating its prevalence and elaborating factors increasing their vulnerability is limited [5, 6]. The South African National Policy Framework developed to address gender-based violence (GBV) in the Post-School Education and Training System has highlighted this critical knowledge gap [6]. Hitherto, research was needed to understand female students’ vulnerability to SV across different higher education institutions in South Africa, including the more resource-poor Technical Vocational Education and Training (TVET) sector. Such research is essential to inform comprehensive policies and SV interventions in South African settings [2, 7].

Research conducted in other countries, including the United States of America and Canada, has shown that the individual characteristics which increase students’ vulnerability to SV include being female, gender-binary non-conformant, coming from poorer community settings, being in the first year of enrolment, being younger, having a disability and exhibiting symptoms of mental ill-health [1, 7–9]. Students with histories of sexual victimisation are more likely to experience SV compared to students without previous experiences [1, 2]. Engaging in high-risk sexual behaviours increases SV vulnerability through interactions with or being targeted by potential perpetrators [1, 2]. Female students who abuse alcohol are more vulnerable to SV, and perpetrators are more likely to attack those who are alcohol-incapacitated [1, 2, 10]. Previous qualitative studies conducted on South African university campuses found similar vulnerability factors and showed the influence of lower socioeconomic status [11–13]. Students from poorer family backgrounds are more likely to engage in sexual risk-taking and transactional sex and become involved in age-disparate and inequitable intimate relationships, all of which are associated with increased vulnerability for SV [11–13].

Ample evidence shows that mental ill-health is an effect of experiencing childhood sexual abuse, other trauma, physical or emotional IPV, partner controlling behaviours, or food insecurity [14–17]. Yet experiences of IPV or other traumas are also associated with prior experiences of childhood sexual abuse [18]. Risky sexual behaviours could be an effect of food insecurity, childhood sexual abuse, or other trauma [18, 19]. Risky sexual behaviours could be associated with mental ill-health, and this relationship may be bi-directional [20–23].

Our study aimed to investigate and describe the inter-relationships of the factors that increase female students’ vulnerability to sexual violence using data collected from nine conveniently selected South African public higher education campuses. Based on existing evidence [1–4, 8, 10–13, 24–26], we hypothesised that experiences of childhood sexual abuse, other life trauma, physical or emotional intimate partner violence (IPV), mental ill-health, harmful alcohol use, food insecurity, and engaging in risky sexual behaviours increase the risk for female students’ experiences of SV. These inter-related vulnerability factors may have direct, indirect, or mediated effects on sexual violence experience.

## Methods

### Study sites

South Africa has two types of public institutions in its higher education system: universities and Technical Vocational Education and Training (TVET) colleges. There are 27 universities and 401 TVET colleges nationally; the latter are found in most communities. We surveyed nine purposefully selected TVET colleges and universities spread across five of South Africa’s nine provinces, namely Gauteng, Eastern Cape, Mpumalanga, KwaZulu Natal, and Limpopo. The research team selected campuses with the input of the National Department of Higher Education and Training (NDHET) and management committees of the selected institutions. Campus selection was purposeful so that we included participants from different demographics and backgrounds, i.e., we ensured that we included students from campuses located near townships, informal settlements, rural and central business districts. None of the selected campuses was known to have notably higher levels of sexual violence compared to non-selected campuses. We conducted the survey between September 2018 and March 2019.

### Sample size calculation

To obtain a self-weighted overall sample size, we calculated the study sample based on the overall female student enrollment in the six TVET colleges and three university campuses. We assumed the prevalence of past-year sexual violence experience amongst TVET and university students of about 15% and 10%, respectively. Therefore, the minimum sample sizes needed to detect the difference in the prevalence between TVET and university students at 5% significance level and 80% power was 565 TVET female students and 840 female university students.

We used different survey marketing strategies, including putting up posters about the survey in public spaces around the campuses, posting on official institutional social media pages, and circulating invites in student WhatsApp groups. The posters had content that included the inclusion criteria for participants and the purpose of the survey, i.e., to "understand young women’s life experiences. The team administered the study over three selected days per campus. The inclusion criteria were female students between the ages of 18–30, who were enrolled in the institutions for at least six months, were willing and available to participate in the survey on selected days. The criterion of at least six months of enrollment at the institution was necessary because the key variable of interest was past year sexual violence experience. In addition, we wanted to include a substantial proportion of participants who could report these experiences while enrolled at their institution. Our final sample consisted of 519 students from the six TVET campuses and 774 students from the three university campuses. This sample size was smaller than expected and resulted from the lower availability of students during the limited data collection period of three days per campus. However, the magnitude of the difference in past-year SV prevalence between TVET and university students in the sample was greater than we had assumed. This gave us 99% power to detect differences in the prevalence of SV between the two groups.

### Questionnaire development and testing

We developed a structured questionnaire using the Research Electronic Data Capture (REDCap) platform [27]. **Table 1** shows variables measured in the survey. We pretested the questionnaire and conducted cognitive testing with fifteen female students enrolled in two campuses that were not part of the primary survey sample. The procedure we followed to pretest the questionnaire was based on previous studies [28]. The cognitive testing process involved administering the questionnaires in short sections. The researchers then conducted group discussions to test for meaning or participant interpretation of what the researchers wanted to know by those questions and probing their feelings after answering the questions. The researchers also explored whether any questions were hard to answer and why [28]. Female students were informed about the purpose of cognitive testing and gave written consent before participating. We used participant feedback to refine and finalise the questionnaire in preparation for data collection in the selected campuses [28]. We loaded the final questionnaire onto electronic devices (tablets), and consenting female students self-completed it. Trained researchers assisted participants in completing the questionnaire when needed.

**Table 1:** Definitions and measurement of variables. Female students survey: South African Technical Vocational Education and Training (TVET) colleges and universities (2018–2019).

Variables	Observed measures	Variable description	Number of items and measurement tool/description	Examples of measurement items.	Cronbach’s alpha
**Outcome** Past year sexual violence	Past year sexual IPV	binary	Three items including forced sex or sexual acts using WHO Domestic Violence Questionnaire [31]	Have you ever had sex with a boyfriend/ husband when you didn’t want to because he physically forced or threatened or pressured you? Did this happen many times, more than once, a few times, once, or did it not happen?	n/a
	Past year non-partner rape	binary	Four items about forced sex by a male non-partner using WHO Domestic Violence Questionnaire [31]	How many times were you forced to have sex by someone who was not your boyfriend/husband? Responses Never, Once, More than one time	
**Observed variable**	Food insecure	binary	Three items on a scale. Food insecure is responding to at least one of the three items with“sometimes” or “often.”	In the past four weeks, did you go a whole day and night without eating anything because there was not enough food? Responses: 0 = Not at all, 1 = Rarely, 2 = Sometimes, 3 = Often	0.77
**Observed variable**	Childhood sexual abuse	score, binary	Six items of the Childhood Trauma Questionnaire [32]	Before I turned 18, I was forced to have sex with a man when I did not want to. Responses: never (= 0), sometimes (= 1), often (= 2), very often (= 3).	0.83
**Latent** Partner violence and controlling behaviors	Past year physical IPV	binary	Five items on experiences of physical IPV using WHO Domestic Violence Questionnaire [31].	Has a boyfriend/husband ever slapped you or thrown something at you which could hurt you? Has any of this happened in the past 12months? Responses Yes/No	n/a
	Past year emotional IPV	binary	Four items on experiences of emotional IPV using WHO Domestic Violence Questionnaire [31].	Has a boyfriend/husband ever insulted you or made you feel bad about yourself? How often has this happened? Has any of this happened in the past 12months? Responses Yes/No	n/a
	Sexual relationship power	score	Eleven items of the South African adaptation of the Sexual Relationship Power Scale (SRPS) [33, 34].	My boyfriend/husband tells me who I can spend time with	0.81
				Responses: Strongly agree (= 4), Agree (= 3), Disagree (= 2), Strongly disagree (= 1)	
**Observed variable**	Other life trauma	Score, +1 binary	Five items of the Life Events checklist [35].	Have you ever experienced any of the following: witnessed the murder of family or friend Possible responses: Yes/No	n/a
**Latent** Mental ill-health	Depressive symptoms	Score, 21+ cut off, binary	Twenty items of the Centre for Epidemiologic Studies Depression (CES-D) Scale [36–38]	bothered by things that usually don’t bother, unable to cheer up even with the help of family or friends Responses: Rarely or none of the time, Some or a little of the time, Moderate amount of time, Most or all of the time	0.88
	PTSD symptoms	Score, 60+ cut off, binary	Thirty items of the Harvard Trauma Questionnaire [38, 39].	experiencing recurrent thoughts or memories of most hurtful or terrifying events, feeling as though the event is happening again, Responses Not at all, A little, Quite A Bit, Extremely	0.96
	Suicidal thoughts	binary	Single question	In the past four weeks, has the thought of ending your life been in your mind? Responses Yes/No	n/a
**Observed** Risky sexual behaviours	Past-year multiple sexual partners	binary	Single question for women who had a sexual partner [40–42]	How many other men are you currently having sex with? Responses: 0, 1, 2,3, 4 or more.	n/a
	Past-year transactional sex	binary	Three items [40–42]	In the past 12 months, have you had sex with anybody because you expected or hoped they would give you money or something else? Responses: Several times; Few times; Once; No	n/a
**Observed variable**	Harmful alcohol use	binary	Three items of the AUDIT C Scale to [43].	How often do you drink five or more alcoholic drinks on the same day? Responses: Never, Occasionally, Monthly, Weekly, Daily, or almost daily	n/a

### Access

We obtained permission to access the institutions and interview female students at different levels. NDHET approved the study and provided inputs into selecting study sites. We also received access from the management of selected universities and TVETs. Each institution provided a contact person who worked closely with the research team and helped publicise the study among the student community.

### Ethics

The South African Medical Research Council’s Human Research Ethics Committee granted ethical approval for the study (EC002-2/2018). Female students were informed about the study and required written consent before participating in the study. They were allocated random and unique study identification numbers and were not required to provide personal details or other personal identifying information to the research team. The use of study IDs ensured the anonymity of the participants. The use of self-administered electronic questionnaires ensured participant privacy during completion. Research assistants synchronised data from the devices to a password-protected, centralised portal and were only accessible to the data manager and project managers [27]. Participants were reimbursed 50 Rands (equivalent US$3.50) for their time and inconvenience. The study was conducted according to the World Health Organization’s Ethical and Safety Recommendations for Research on Domestic Violence against Women. The trained research assistants took care to ensure participant confidentiality and to minimise participant distress [29, 30]. They also gave participants referral information about the GBV focused and psychosocial support services within their campuses and surrounds.

### Data analysis

We conducted the analysis using Mplus 6 Software [44, 45]. We examined the extent of missing data due to non-response and found less than 5% missing data in some variables. However, none of the participants had missing data on the outcome variables; therefore, we included data from all the participants in the analysis. We combined the co-related indicators of risky sexual practices—transactional sex and having multiple concurrent sexual partners—into a binary variable with the categories being (0) no transactional sex or multiple sexual partners versus (1) any transactional sex or multiple sexual partners. We carried out descriptive analysis by using cross-tabulations. We used the binary past year SV outcome in conducting logistic regression to test the associations between variables.

We conducted confirmatory factor, convergent, and discriminant validity analysis through the following steps [46]. First, we created measurement models for two latent constructs: (1) mental ill-health consisting of depressive symptoms, PTSD, and suicidal thoughts; and (2) the physical or emotional IPV and partner controlling behaviours. Second, we estimated each of the latent factor measurement models separately. Third, we assessed latent factor loadings and fit indices, namely the Comparative fit Index (CFI), Tucker-Lewis Index (TLI), and Root Mean Square Error of Approximation (RMSEA). Fourth, we assessed the convergent and discriminant validity of each of the latent factors using Average Variance Extracted (AVE) >0.05 and Discriminant validity or squared correlations of the two constructs <0.85 [47, 48].

**Fig 1:**
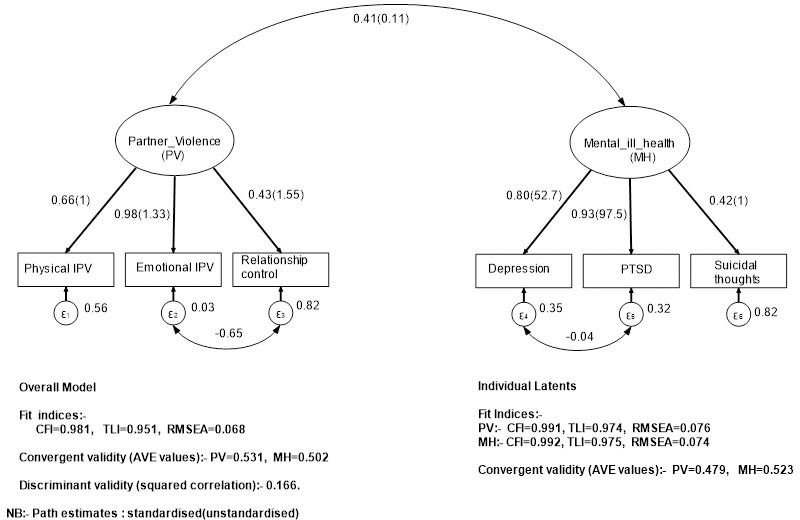
Latent convergent and discriminant validity model (female students survey: South African Technical Vocational Education and Training (TVET) colleges and universities (2018–2019).

**Fig 1** summarises the two latent constructs, their factor loadings, reliability coefficients, and the corresponding model fit statistics. We used the criteria of CFI and TLI >0.90 as indicative of fair fit; CFI and TLI >0.95 as good fit; and RMSEA ranging 0.05–0.1 as acceptable< fit and RMSEA <0.05 as good fit [49, 50]. Based on these criteria, the two latent factors had high factor loadings and good fit indices. In addition, the convergent statistics (AVE) for the two latents were higher than the discriminant ones (squared correlations), which was indicative of good discriminant validity.

Following the confirmatory factor analyses, we performed a full model path analysis to assess the paths between the past year SV outcome, the two latent constructs, and observed factors as per our apriori hypothesised model [51]. Based on existing literature, we hypothesised that: 1. Childhood sexual abuse experience directly impacts SV experience and through risky sexual behaviour; 2. Other life trauma impacts SV experience through alcohol use, mental ill-health, and partner violence experience; 3. Alcohol use impacts SV experience through risky sexual behaviour; 4. Food insecurity impacts SV experience through mental ill health; 5. Risky sexual behaviour impacts mental ill health through partner violence experience; 6. Other life trauma directly impacts mental ill-health and through partner violence; 7. Childhood SV experience directly impacts mental ill-health and through partner violence; 8. Food insecurity impacts mental ill-health directly and through partner violence experience; 9. Childhood sexual abuse impacts partner violence experience directly and through risky sexual behaviour; 10. Alcohol use directly impacts partner violence experience through risky sexual behaviour; 11. Other life trauma impacts IPV directly and through alcohol use; 12. Food insecurity impacts SV through risky sexual behaviour; 13. Risky sexual behaviour impacts SV experience; 14. Mental ill-health impacts SV.

We built a Generalised Structural Equation Model (GSEM) that depicted the apriori-hypothesised paths. We estimated models inductively using weighted least squares mean and variance estimators (WLSMV) to deal with missing data. We undertook iterative model modifications, including deleting non-significant paths, which we conceptualised but were unproven until we obtained a theoretically sound model comprising only statistically significant pathways. We used modification indices to improve the model fit and allowed errors of variables to co-vary when it was theoretically justifiable. We tested the model’s goodness of fit using CFI> = 0.9 and TLI > = 0.9 and RMSEA< = 0.08 as indicative of acceptable fit [48–50].

## Results

**Table 2** shows the prevalence of SV and sample characteristics disaggregated by type of higher education institution. The sample comprised 1293 participants: 40% were enrolled in TVETs, and 60% were enrolled in universities. Most of the participants were in the 18-24-year age group (82.9%). Forty-two percent of the participants were food insecure, and the difference in proportion between the two types of institutions was not statistically significant. Eighty percent of the participants were in heterosexual intimate relationships at the time of the study.

**Table 2:** Prevalence of past-year SV, socio-demographic characteristics and relationship status of participants disaggregated by institution. Female students survey: South African Technical Vocational Education and Training (TVET) colleges and universities (2018–2019).

	All	TVET colleges	Universities							
	N	n/ mean	%/ sd	N	n/ mean	%/ sd	N	n/ mean	%/ sd	p-value
**Past year SV** (sexual IPV & non-partner rape)	1293	260	20.1	519	141	27.2	774	119	15.4	<0.001
**Past year non-partner rape experience**	1293	97	7.5	519	52	10	774	45	5.8	0.005
**Past year sexual IPV**	1233			487			746			
No		1004	81.4		363	74.5		641	85.9	<0.001
Yes		210	17.0		122	25.1		88	11.8	
*Missing info*		19	1.6		2	0.4		17	2.3	
**Age group†**	1293			519			774			
18–24		1072	82.9		410	79.0		662	85.5	<0.001
25–30		200	15.5		92	17.7		108	14.0	
*Missing info*		21	1.6		17	3.3		4	0.5	
**Food insecure**	1293	548	42.4	519	217	41.8	774	331	42.8	0.734
**Currently in heterosexual intimate relationship**	1233	1031	83.6	487	464	95.3	746	567	76.0	<0.001

TVET: Technical and Vocational Education and Training, IPV: Intimate partner violence.

Twenty percent of the 1293 participants experienced past year partner SV and or non-partner rape: 7.5% (97/1293) experienced non-partner rape, and 17% (210/1233) experienced partner sexual violence. A significantly higher proportion of TVETs participants (27% of 519) reported past-year SV experiences compared to university participants (15% of 774) (p<0.001).

### Factors associated with SV in the past year

**Table 3** shows factors associated with participants’ SV experiences from bivariate and multivariate logistic regression analysis. In multivariate analysis, university participants were less likely to experience past year SV compared to TVET participants. Participants who experienced childhood sexual abuse were more likely to experience past year SV compared to those who did not experience childhood sexual abuse. Those who experienced physical or emotional partner violence were four times more likely to report past-year SV compared to those whose partners did not abuse them. The risk of past-year SV increased with the partner controlling behaviour scores. Participants who engaged in risky sexual behaviours were thrice more likely to experience past year SV compared to those who did not engage in risky sexual behaviours. Participants who had mental ill-health symptoms, i.e., depressive, PTSD symptoms, and suicidal thoughts, were twice more likely to have experienced past-year SV experience. Harmful alcohol use was associated with increased risk for past year SV. Other life trauma was associated with past-year SV experience in bivariate analysis but not in multivariate analysis.

**Table 3:** Associations between female students’ past year SV experiences and vulnerability factors. Female students survey: South African Technical Vocational Education and Training (TVET) colleges and universities (2018–2019).

	All participants (N = 1293)			No past year SV (N = 1033)		Past year SV (N = 260)					
Vulnerability Factors	N	n/ mean	%†/sd	n/ mean	%† /sd	n/ mean	%† /sd	OR (95%CI)	p-value	aOR (95%CI) ^b^	p-value
**Food insecurity score(high = insecure) ‡**	1293	2.0	2.2	1.9	2.1	2.3	2.2	1.08(1.02–1.15)	0.010	1.08(1.02–1.15)	0.014
**Institution(n = 1293)**											
**TVET college**		519	40.0	378	36.6	141	54.2	ref		ref	
**University**		774	60.0	655	63.4	119	45.8	0.49(0.37–0.64)	<0.001	0.48(0.37–0.64)	0.001
**Residence(n = 1277)**											
**On-campus**		627	49.1	508	49.7	119	46.7	ref		ref	
**Off-campus**		650	50.9	514	50.3	136	53.3	1.13(0.86–1.49)	0.385	1.14(0.87–1.51)	0.345
**Childhood sexual abuse experience score (high = more abuse) ‡**	1293	590	45.6	1.2	2.4	2.4	3.1	1.15(1.09–1.22)	<0.001	1.15(1.09–1.21)	<0.001
*Past year IPV experience*											
**Experienced Emotional IPV**	1216	413	34.0	127	13.2	106	41.7	4.27(3.20–5.71)	<0.001	4.26(3.18–5.71)	<0.001
**Experienced Physical IPV**	1216	233	19.2	258	26.8	155	61.0	4.71(3.45–6.43)	<0.001	4.64(3.39–6.35)	<0.001
**Sexual relationship power and partner controlling behaviour score (high = partner more controlling) ‡**	1266	20.7	5.5	20.1	5.2	23.3	6.1	1.11(1.08–1.14)	<0.001	1.11(1.08–1.14)	<0.001
**Other trauma events (Witnessed murder, hijacking or kidnapping)**	1275	268	21.0	202	19.8	66	25.7	1.40(1.01–1.92)	0.040	1.29(0.93–1.79)	0.126
*Mental health*											
**Depressive symptoms (CESD 21+)**	1235	535	43.3	391	39.5	144	58.5	2.16(1.63–2.87)	<0.001	2.14(1.61–2.85)	<0.001
**PTSD symptoms (HTQ 61+)**	1293	90	7	55	5.3	35	13.5	2.77(1.77–4.33)	<0.001	2.80(1.79–4.38)	<0.001
**Suicidal thoughts**	1269	267	21.0	189	18.7	78	30.5	1.91(1.40–2.60)	<0.001	1.84(1.34–2.51)	<0.001
**Risky sexual behaviours “Multiple partners and/or transactional sex**	1293	1062	82.1	820	82.1	242	93.1	3.54(2.08–6.02)	<0.001	3.83(2.21–6.65)	<0.001
**Past year harmful alcohol use**		448	34.7	333	33.6	115	45.6	1.66(1.25–2.19)	<0.001	1.61(1.21–2.14)	0.001

‡: Continuous exposures.

†: All are column percentages (All participants, N = 1293; No past year SV, n = 1033; past-year SV, n = 260). TVET: Technical and Vocational Education and Training, IPV: Intimate partner violence, CESD: Center for Epidemiologic Studies Depression Scale; HTQ: Harvard Trauma questionnaire, PTSD: Posttraumatic stress disorder. ^b:^ Model adjusted for age.

**Fig 2:**
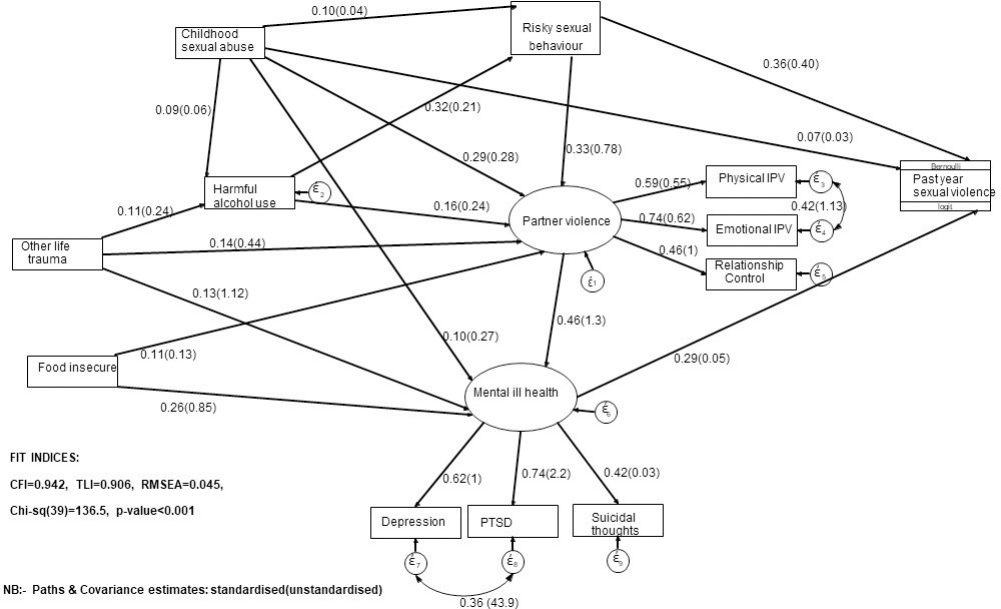
Structural pathways between SV experiences and inter-related vulnerability factors (female students survey: South African Technical Vocational Education and Training (TVET) colleges and universities (2018–2019)).

**Fig 2** shows the final GSEM model. The GSEM model had fair fit (CFI = 0.942, TLI = 0.906 RMSEA = 0.045).

**Table 4** shows the GSEM output, including the latent constructs’ direct, indirect, and total effects and observed factors on the SV latent outcome. Childhood sexual abuse had direct and indirect effects on past year SV mediated by risky sexual behaviours. Mental ill-health also mediated the indirect effects of childhood sexual abuse on past year SV. Childhood sexual abuse had direct and indirect effects on physical, emotional partner violence or controlling behaviours mediated by harmful alcohol use. Risky sexual behaviours mediated the indirect effects of childhood sexual abuse on physical, emotional partner violence, or controlling behaviours. Risky sexual behaviours mediated the indirect effects of harmful alcohol use on past year SV. Harmful alcohol use had direct and indirect effects on physical, emotional partner violence or controlling behaviours, also mediated by risky sexual behaviours. Other life trauma had indirect effects on past year SV mediated by mental ill-health. Other life trauma, directly and indirectly, affected physical and emotional partner violence or controlling behaviours mediated by harmful alcohol use. Food insecurity had indirect effects on past year SV, which were mediated by mental ill-health. Food insecurity had direct and indirect effects on mental ill-health mediated by physical, emotional partner violence or controlling behaviours. Mental ill-health also mediated the indirect effects of physical and emotional partner violence or controlling behaviours on the past year SV.

**Table 4:** Generalised structural model statistics. Female students survey: South African Technical Vocational Education and Training (TVET) colleges and universities (2018–2019).

	Direct Effects			Total Indirect Effects			Total Effects		
	Unstd Coef. (95%CI)	Std Coef	p-value	Unstd Coef. (95%CI)	Std Coef	p-value	Unstd Coef. (95%CI)	Std Coef	p-value
Past year SV ← Childhood sexual abuse	0.03(0–0.07)	0.073	0.044	0.06(0.03–0.08)	0.121	<0.00l	0.09(0.06–0.12)	0.194	<0.00l
Past year SV ← Other life trauma				0.11(0.06–0.15)	0.071	<0.00l	0.11(0.06–0.15)	0.071	<0.00l
Past year SV ← Harmful alcohol use				0.11(0.07–0.14)	0.15	<0.00l	0.11(0.07–0.14)	0.15	<0.00l
Past year SV ← Food insecure				0.05(0.03–0.07)	0.089	<0.00l	0.05(0.03–0.07)	0.089	<0.00l
Mental ill-health ← Risky sexual behaviour				0.99(0.63–1.36)	0.152	<0.00l	0.99(0.63–1.36)	0.152	<0.00l
Mental ill health ← Other life trauma	1.12(0.52–1.71)	0.13	<0.00l	0.68(0.37–0.98)	0.077	<0.00l	1.79(1.19–2.39)	0.203	<0.00l
Mental ill health ← Childhood sexual abuse	0.27(0.11–0.43)	0.1	<0.001	0.43(0.31–0.55)	0.158	<0.00l	0.7(0.52–0.87)	0.258	<0.00l
Mental ill health ← Food insecure	0.85(0.64–1.07)	0.26	<0.00l	0.16(0.06–0.26)	0.049	0.002	1.01(0.78–1.25)	0.311	<0.00l
Partner violence and controlling behaviour ← Childhood sexual abuse	0.28(0.21–0.35)	0.286	<0.00l	0.06(0.02–0.09)	0.057	0.001	0.34(0.26–0.41)	0.344	<0.00l
Partner violence and controlling behaviour ← Harmful alcohol use	0.24(0.12–0.35)	0.157	<0.00l	0.16(0.09–0.23)	0.105	<0.00l	0.40(0.29–0.51)	0.262	<0.00l
Partner violence and controlling behaviour ← Other life trauma	0.44(0.24–0.64)	0.137	<0.00l	0.09(0.04–0.14)	0.029	<0.00l	0.53(0.32–0.75)	0.166	<0.00l
Past year SV ← Partner violence and controlling behaviour				0.06(0.03–0.09)	0.132	<0.00l	0.06(0.03–0.09)	0.132	<0.00l
Past year SV ← Mental ill health	0.05(0.03–0.07)	0.29	<0.001				0.05(0.03–0.07)	0.29	<0.001
Past year SV ← Risky sexual behaviour	0.40(0.26–0.54)	0.36	<0.001				0.40(0.26–0.54)	0.40	<0.001
Harmful alcohol use ← Other life trauma	0.24(0.13–0.34)	0.11	<0.001				0.24(0.13–0.34)	0.11	<0.001
Harmful alcohol use ← Childhood sexual abuse	0.06(0.02–0.1)	0.09	<0.001				0.06(0.02–0.1)	0.09	<0.001
Mental ill-health ← Harmful alcohol use				0.51(0.33–0.68)	0.121	<0.00l	0.51(0.33–0.68)	0.121	<0.00l
Mental ill health ← Partner violence and controlling behaviour	1.27(0.97–1.58)	0.46	<0.001				1.27(0.97–1.58)	0.46	<0.001
Partner violence and controlling behaviour ← Risky sexual behaviour	0.78(0.51–1.06)	0.33	<0.001				0.78(0.51–1.06)	0.33	<0.001
Partner violence and controlling behaviour ← Food insecure	0.13(0.05–0.2)	0.105	<0.00l				0.13(0.05–0.2)	0.105	0.001
Partner violence and controlling behaviour ← Harmful alcohol use	0.24(0.12–0.35)	0.157	<0.00l	0.16(0.09–0.23)	0.105	<0.00l	0.40(0.29–0.51)	0.262	<0.00l
Risky sexual behaviour ← Harmful alcohol use	0.21(0.16–0.25)	0.32	<0.001				0.21(0.16–0.25)	0.32	<0.001
Risky sexual behaviour ← Childhood sexual abuse	0.04(0.01–0.07)	0.101	0.008	0.01(0–0.02)	0.029	0.003	0.05(0.02–0.09)	0.13	0.001

**Table 5** compares the apriori-hypotheses with the summary of findings from our data. Notably, the apriori-hypothesis that food insecurity impacts SV through risky sexual behaviour was not proven by our data, i.e., we found no significant paths or effects.

**Table 5:** Summary comparison of apriori-hypothesis and findings. Female students survey: South African Technical Vocational Education and Training (TVET) colleges and universities (2018–2019).

	Hypothesis	Significant direct effects	Significant indirect effects	Specific indirect effect	Unstd Coef.	Std Coef	p-value
					(95% CI)		
1	Childhood sexual abuse experience directly impacts SV experience and through risky sexual behaviour	√	√	SV← RB←CSA	0.03(0.01–0.04)	0.057	0.002
2	Other life trauma impacts SV experience through alcohol use, mental ill-health, and partner violence experience	-	√	SV ←MH←TRA	0.05(0.02–0.08)	0.033	0.004
				SV← RB← ALC← TRA	0.02(0.01–0.03)	0.014	0.001
				SV←MH← PV← TRA	0.02(0.01–0.04)	0.016	0.004
3	Alcohol use impacts SV experience through risky sexual behaviour	-	√	SV← RB ←ALC	0.09(0.05–0.12)	0.122	<0.001
4	Food insecurity impacts SV experience through mental ill-health	-	√	SV← MH ← FDI	0.04(0.02–0.06)	0.069	<0.001
5	Risky sexual behaviour impacts mental ill health through partner violence experience	-	√	MH ←PV ← RB	1.12(0.73–1.51)	0.159	<0.001
6	Other life trauma directly impacts mental ill-health and through partner violence	√	√	MH ←PV← TRA	0.58(0.29–0.87)	0.06	<0.001
7	Childhood SV experience directly impacts mental ill-health and through partner violence	√	√	MH←PV← CSA	0.35(0.24–0.45)	0.117	<0.001
8	Food insecurity impacts mental ill-health directly and through partner violence experience	√	√	MH← PV←FDI	0.17(0.06–0.27)	0.047	0.002
9	Childhood sexual abuse impacts partner violence experience directly and through risky sexual behaviour	√	√	PV← RB ← CSA	0.05(0.02–0.09)	0.054	0.002
10	Alcohol use directly impacts partner violence experience, and through risky sexual behaviour	√	√	PV← RB ← ALC	0.18(0.1–0.25)	0.116	<0.001
11	Other life trauma impacts partner violence experience directly, and through alcohol use	√	√	PV← ALC← TRA	0.06(0.02–0.09)	0.017	0.004
				PV ←RB ←ALC ←TRA	0.04(0.02–0.07)	0.013	0.001
12	Food insecurity impacts SV through risky sexual behaviour	-	-	-	-	-	-
13	Risky sexual behaviour impacts SV experience	√	-	n/a	n/a	n/a	n/a
14	Mental ill-health impacts SV	√	-	n/a	n/a	n/a	n/a

MH = Mental ill health; SV = Past year SV; RB = Risky sexual behaviour; PV = Partner violence and controlling behaviour; CSA = Childhood sexual abuse; TRA = Other life trauma; ALC = Harmful alcohol use; FDI = Food insecure Unstd Coef. = Unstandardised Coefficient; Std Coef. = Standardised Coefficient.

## Discussion

Our study confirmed the continued occurrence of female students’ sexual victimisation in the South African Higher education setting. Moreover, the findings show multiple and inter-related characteristics were associated with increased vulnerability. TVET college participants were more vulnerable, and they reported a higher prevalence of past-year SV experience compared to university participants. The data confirms the inter-relatedness of vulnerability factors that impacts female students’ experiences of SV. The long-term impacts of childhood sexual abuse on SV experiences were evident. However, some of the effects were indirect and mediated by other amenable factors, namely risky sexual behaviors, physical or emotional partner violence, controlling behaviours, and mental ill-health. Engaging in risky sexual behaviours had effects on past year SV. Mental ill-health, which was associated with the cumulative impacts of other traumatic exposures, risky sexual behaviours, inequitable and abusive intimate relationships, and food insecurity, increased vulnerability to past year SV experience.

Our findings are consistent with literature that suggests that childhood sexual abuse increases vulnerability for adult sexual assault through different pathways, including impacts on risk perceptions, sexual risk-taking, and behavioural responses [1, 2, 4, 24, 52]. The impacts of traumatic exposures and childhood sexual abuse on adult harmful alcohol use, the role of alcohol intoxication in reducing sexual assault risk recognition, and increasing sexual victimisation are well documented [1, 2, 4, 10, 14, 52]. Engaging in risky behaviours also indirectly impacted mental health, consistent with previous research that indicated the clustering of harmful alcohol use and depressive symptoms among youths who engage in risky sexual behaviours [2, 20, 22, 53]. Risky behaviours also impacted partner violence or controlling behaviours, as shown in other studies [18, 41]. Our findings also confirmed the cumulative effects of traumatic, violent exposures and food insecurity on mental ill-health that have been demonstrated in global literature [14–16, 54, 55].

Moreover, we have shown that mental ill-health directly impacts SV experience. For example, PTSD, depression, and harmful alcohol use alter women’s judgment about sexual assault risk making them less likely to practice self-protective behaviours. In addition, PTSD, depression, and harmful alcohol use increase women’s vulnerability as "easy targets to potential perpetrators [2, 4, 52]. Overall, this data shows the complex relationships of mental ill-health as both an effect and risk factor for women’s experiences of violence.

The indirect effects of food insecurity on physical, emotional partner violence or controlling behaviours and past year SV is consistent with existing literature from other settings, which shows that women who live in circumstances of deprivation have diminished power in their main sexual relationships [19, 21, 41, 56–58]. Women are thus vulnerable within contexts of inequitable or age-disparate relationships, economic dependency, multiple sexual partners, and when they engage in transactional sex [11, 19, 21, 41, 56–61]. In addition, previous South African studies showed the impacts of food insecurity and poverty on mental ill-health and victimisation [14, 16, 60–63].

Men perpetrate SV against women. Therefore, its prevention must involve interventions targeted at men, which are vital within South African higher education settings. Nevertheless, our research focused on factors that increased female students’ vulnerability to past year SV experiences in South African higher education settings. We have shown that the factors associated with female students’ greater vulnerability to victimisation are inter-related. Scholars have effectively reduced female students’ vulnerability to SV in the global North by implementing multi-component interventions that address mental ill-health, risky sexual behaviours, and gender inequitable relationships [64, 65]. In our study, risky sexual behaviours had strong associations with past-year SV, therefore, there is a need to adapt and test interventions that focus on equipping female students with skills to recognise and resist sexual assault on South African campuses. Mental ill-health was a central mediator of the relationships of other vulnerability factors and past year SV. Therefore, interventions addressing it may potentially reduce SV risk. Psychosocial support and trauma-focused therapies are essential to help students heal from the mental-ill health effects of previous traumatic exposures and alleviate future victimisation [66–69]. South African higher education institutions must administer SV prevention interventions and provide survivor-friendly psychosocial services. Mental ill-health was associated with socioeconomic pressures and food insecurity. Therefore, it is essential to implement interventions that reduce vulnerability to SV by equipping female students with financial management skills and building their resilience to withstand social, material pressures or other life stressors [70]. Our study found strong associations between physical, emotional partner violence or controlling behaviours and past year SV. Thus, implementing gender-transformative interventions which empower female students with communication skills to express themselves better and assert their rights within intimate relationships is imperative for these settings [64, 70–73].

The study has several limitations. Firstly, our study used a cross-sectional design. Therefore, it has limitations in establishing the temporality of variables and causality. However, by using variables or measures that were restricted in time, we could assume the directionality of associations of variables as applied in the GSEM. Most importantly, the terms "effects" or "impacts" used in reporting and interpreting GSEM output, because of the use of cross-sectional data, relate to the directionality of the variables as illustrated by the path arrows and do not imply causality [50]. Secondly, we measured variables across the lifetime and more recently in the past 12months. Therefore, there is the possibility for recall bias for the factors measured earlier in life, particularly experiences of childhood abuse, may be underestimated due to failure to recall experiences. Thirdly, our study is limited in generalisability because we recruited participants through a convenience sampling strategy. Further research is needed using a representative sample to truly reflect levels of past-year SV among female students enrolled in South African Universities and TVET colleges. Finally, we conducted data collection in each campus over a limited duration of three days. Some students who had classes or other pressing academic commitments on the data collection days may have been excluded. Selection bias in which students who responded to research adverts are different from those who did not respond is possible. For example, students residing on the campus may have had greater participation chances compared to students who lived off-campus who may not have come to the campuses while the survey was ongoing. Notwithstanding, we assumed that these biases in recruitment were equal across the different settings and would impact prevalence more than the relationship between variables. We are confident that the insights we have collated are of interest to the South African higher education sector and other low- and middle-income country (LMICs) settings, considering the limited empirical data to inform response and programming.

## Conclusion

Our study has highlighted the pervasive SV experienced by female students in South African higher education settings. We found that female students’ past year experiences of SV were associated with amenable factors, which included risky sexual behaviours, mental ill-health symptoms, the pervasive climate of gender inequality and violence against women within intimate relationships, and food insecurity. There is a critical need for comprehensive, multi-dimensional interventions that have the necessary elements to effectively reduce women’s vulnerability to SV through addressing the multiple, inter-related risk factors that we have identified. The South African higher education sector must prioritise research to develop, test, and scale effective SV interventions. Given that our study implicated childhood sexual abuse for increasing SV vulnerability among female students, society-wide interventions must be implemented to prevent young girls’ sexual victimization within South African communities.

## Supporting information

S1 File (CSV)

## References

[1] Calhoun KS, Mouilso ER, Edwards KM. Sexual assault among college students. In: McAnulty RD, editor. Sex in college: The things they don’t write home about. Sex, Love and Psychology. Santa Barbra, California. Denver,Colorado. Oxford, England.: ABC-Clio, LLC; 2012. p. 263–88. 10.5860/choice.50-1773.

[2] Edwards KM, Sessarego SN. Chapter 3—Risk of Rape Across the Ecosystem: Outlining a Framework for Sexual Assault Risk Reduction and Resistance Education. In: Orchowski LM, Gidycz CA, editors. Sexual Assault Risk Reduction and Resistance. San Diego: Academic Press; 2018. p. 39–66. 10.1016/B978-0-12-805389-8.00003-7.

[3] Fielding-Miller R, Shabalala F, Masuku S, Raj A. Epidemiology of campus sexual assault Among University women in Eswatini. Journal of interpersonal violence. 2019. 10.1177/0886260519888208 31738110 PMC7231640

[4] Jewkes R, Sen P, Garcia-Moreno C. Sexual violence in: World report on violence and health. Geneva: World Health Organization. 2002;26. 10.1007/bf03405037.

[5] Gouws A. # EndRapeCulture Campaign in South Africa: Resisting Sexual Violence Through Protest and the Politics of Experience. Politikon. 2018;45(1):3–15. 10.1080/02589346.2018.1418201.

[6] South African Department of Higher Education and Training n. Policy Framework to address Gender-Based Violence in the Post-School Education and Training System version 4 for Public Comment. In: Training DoHEa, editor. Pretoria2019.

[7] Bonar EE, DeGue S, Abbey A, Coker AL, Lindquist CH, McCauley HL, et al. Prevention of sexual violence among college students: Current challenges and future directions. Journal of American College Health. 2020:1–14. 10.1080/07448481.2020.1757681 32407244 PMC7666108

[8] Senn CY, Eliasziw M, Barata PC, Thurston WE, Newby-Clark IR, Radtke HL, et al. Sexual violence in the lives of first-year university women in Canada: no improvements in the 21st century. BMC Women’s Health. 2014;14(1):135. doi: 10.1186/s12905-014-0135-4 25410412 PMC4228092

[9] Orchowski LM, Gidycz C. Sexual Assault Risk Reduction and Resistance: Theory, Research, and Practice: Academic Press; 2018.

[10] Abbey A. Alcohol-related sexual assault: A common problem among college students. Journal of Studies on Alcohol, supplement. 2002(14):118–28. 10.15288/jsas.2002.s14.118 12022717 PMC4484270

[11] Clowes L, Shefer T, Fouten E, Vergnani T, Jacobs J. Coercive sexual practices and gender-based violence on a university campus. Agenda. 2009;23(80):22–32.

[12] Shefer T, Clowes L, Vergnani T. Narratives of transactional sex on a university campus. Culture, health & sexuality. 2012;14(4):435–47. doi: 10.1080/13691058.2012.664660 22390385

[13] Hames M. “Let us burn the house down!” Violence against women in the higher education environment. Agenda. 2009;23(80):42–6.

[14] Devries KM, Child JC, Bacchus LJ, Mak J, Falder G, Graham K, et al. Intimate partner violence victimization and alcohol consumption in women: a systematic review and meta-analysis. Addiction. 2014;109(3):379–91. 10.1111/add.12393 24329907

[15] World Health Organization. Social determinants of mental health. Geneva: Switzerland: World Health Organization; 2014.

[16] Devries KM, Mak JY, Bacchus LJ, Child JC, Falder G, Petzold M, et al. Intimate partner violence and incident depressive symptoms and suicide attempts: a systematic review of longitudinal studies. PLoS medicine. 2013;10(5):e1001439. 10.1371/journal.pmed.1001439 23671407 PMC3646718

[17] World Health Organization. Global and regional estimates of violence against women: prevalence and health effects of intimate partner violence and non-partner sexual violence. Geneva: World Health Organization; 2013.

[18] Messersmith LJ, Halim N, Steven Mzilangwe E, Reich N, Badi L, Holmes NB, et al. Childhood trauma, gender inequitable attitudes, alcohol use and multiple sexual partners: correlates of intimate partner violence in northern Tanzania. Journal of interpersonal violence. 2017:0886260517731313. 10.1177/0886260517731313. 29294914

[19] Wellings K, Collumbien M, Slaymaker E, Singh S, Hodges Z, Patel D, et al. Sexual behaviour in context: a global perspective. The Lancet. 2006;368(9548):1706–28. 10.1016/s0140-6736(06)69479-8 17098090

[20] Donovan C, McEwan R. A review of the literature examining the relationship between alcohol use and HIV-related sexual risk-taking in young people. Addiction. 1995;90(3):319–28. doi: 10.1111/j.1360-0443.1995.tb03780.x 7735017

[21] Wamoyi J, Stobeanau K, Bobrova N, Abramsky T, Watts C. Transactional sex and risk for HIV infection in sub-Saharan Africa: a systematic review and meta-analysis. Journal of the international AIDS society. 2016;19(1):20992. 10.7448/ias.19.1.20992 27809960 PMC5095351

[22] Kalichman SC, Simbayi LC, Kaufman M, Cain D, Jooste S. Alcohol use and sexual risks for HIV/AIDS in sub-Saharan Africa: systematic review of empirical findings. Prevention science. 2007;8(2):141. 10.1007/s11121-006-0061-2 17265194

[23] Watt MH, Ranby KW, Meade CS, Sikkema KJ, MacFarlane JC, Skinner D, et al. Posttraumatic stress disorder symptoms mediate the relationship between traumatic experiences and drinking behavior among women attending alcohol-serving venues in a South African township. Journal of studies on alcohol and drugs. 2012;73(4):549–58. 10.15288/jsad.2012.73.549 22630793 PMC3364321

[24] Polusny MA, Follette VM. Long-term correlates of child sexual abuse: Theory and review of the empirical literature. Applied and preventive psychology. 1995;4(3):143–66. 10.1016/s0962-1849(05)80055-1.

[25] Messman TL, Long PJ. Child sexual abuse and its relationship to revictimization in adult women: A review. Clinical Psychology Review. 1996;16(5):397–420. 10.1016/0272-7358(96)00019-0.

[26] Abrahams N, Devries K, Watts C, Pallitto C, Petzold M, Shamu S, et al. Worldwide prevalence of non-partner sexual violence: a systematic review. The Lancet. 2014;383(9929):1648–54. 10.1016/s0140-6736(13)62243-6 24529867

[27] Patridge EF, Bardyn TP. Research electronic data capture (REDCap). Journal of the Medical Library Association: JMLA. 2018;106(1):142. 10.5195/jmla.2018.319.

[28] Sikweyiya Y, Jewkes R, Morrell R. Talking about rape: South African men’s responses to questions about rape. Agenda. 2007;21(74):48–57.

[29] Ellsberg M, Heise L, Pena R, Agurto S, Winkvist A. Researching domestic violence against women: methodological and ethical considerations. Studies in family planning. 2001;32(1):1–16. 10.1111/j.1728-4465.2001.00001.x 11326453

[30] Jewkes R, Watts C, Abrahams N, Penn-Kekana L, Garcia-Moreno C. Ethical and methodological issues in conducting research on gender-based violence in Southern Africa. Reproductive health matters. 2000;8(15):93–103. 10.1016/s0968-8080(00)90010-7 11424273

[31] World Health Organization. WHO multi-country study on women’s health and domestic violence: Core questionnaire and WHO instrument-Version 9. Geneva: World Health Organization. 2000;913.

[32] Bernstein DP, Stein JA, Newcomb MD, Walker E, Pogge D, Ahluvalia T, et al. Development and validation of a brief screening version of the Childhood Trauma Questionnaire. Child abuse & neglect. 2003;27(2):169–90. 10.1097/00005053-199202000-00008 12615092

[33] Jewkes R, Nduna M, Jama P, Levin J, editors. Measuring relationship power: adaptation of the SRPS for South Africa. XIV International AIDS Conference; Barcelona, Spain; 2002.

[34] Pulerwitz J, Gortmaker SL, DeJong W. Measuring sexual relationship power in HIV/STD research. Sex roles. 2000;42(7):637–60. 10.1023/a:1007051506972.

[35] Weathers F, Blake D, Schnurr P, Kaloupek D, Marx B, Keane T. The life events checklist for DSM-5 (LEC-5). Instrument available from the National Center for PTSD at www ptsd va gov [Internet]. 2013.

[36] Radloff LS. The CES-D scale: A self-report depression scale for research in the general population. Applied psychological measurement. 1977;1(3):385–401. 10.1177/014662167700100306.

[37] Pretorius TB. Cross-cultural application of the Center for Epidemiological Studies depression scale: a study of black South African students. Psychological Reports. 1991;69:1179–85. 10.2466/pr0.1991.69.3f.1179 1792288

[38] Rothon C, Stansfeld SA, Mathews C, Kleinhans A, Clark C, Lund C, et al. Reliability of self report questionnaires for epidemiological investigations of adolescent mental health in Cape Town, South Africa. Journal of Child & Adolescent Mental Health. 2011;23(2):119–28. 10.2989/17280583.2011.634551 25860086

[39] Mollica RF, Caspi-Yavin Y, Bollini P, Truong T, Tor S, Lavelle J. The Harvard trauma questionnaire. Journal of Nervous and Mental Disease. 1992;180(2):111–6. 10.1037/t07469-000. 1737972

[40] Dunkle KL, Jewkes R, Nduna M, Jama N, Levin J, Sikweyiya Y, et al. Transactional sex with casual and main partners among young South African men in the rural Eastern Cape: prevalence, predictors, and associations with gender-based violence. Social science & medicine. 2007;65(6):1235–48. 10.1016/j.socscimed.2007.04.029. 17560702 PMC2709788

[41] Dunkle KL, Jewkes RK, Brown HC, Gray GE, McIntryre JA, Harlow SD. Transactional sex among women in Soweto, South Africa: prevalence, risk factors and association with HIV infection. Social science & medicine. 2004;59(8):1581–92. 10.1016/j.socscimed.2004.02.003 15279917

[42] Chawla N, Sarkar S. Defining “high-risk sexual behavior” in the context of substance use. Journal of Psychosexual Health. 2019;1(1):26–31. 10.1177/2631831818822015.

[43] Saunders JB, Aasland OG, Babor TF, De la Fuente JR, Grant M. Development of the alcohol use disorders identification test (AUDIT): WHO collaborative project on early detection of persons with harmful alcohol consumption-II. Addiction. 1993;88(6):791–804. 10.1111/j.1360-0443.1993.tb02093.x 8329970

[44] Muthén L, Muthén B. Mplus user’s guide 6th edition. Los Angeles, California: Muthen & Muthen. 2010.

[45] Wang J, Wang X. Structural equation modeling: Applications using Mplus: John Wiley & Sons; 2019.

[46] Brown TA, Moore MT. Confirmatory factor analysis. Handbook of structural equation modeling. 2012:361–79. doi: 10.1007/s11682-012-9190-3 22777078 PMC3538867

[47] Mehmetoglu M, Jakobsen TG. Applied statistics using Stata: a guide for the social sciences: Sage; 2016.

[48] Hair J, Black W, Babin B, Anderson R. Multivariate Data Analysis (S. Edition Ed.). United State of America: Pearson Education Limited. 2014.

[49] Lt Hu, Bentler PM. Cutoff criteria for fit indexes in covariance structure analysis: Conventional criteria versus new alternatives. Structural equation modeling: a multidisciplinary journal. 1999;6(1):1–55.

[50] Awang Z. Research methodology and data analysis second edition: UiTM Press; 2012.

[51] Wang J, Wang X. Structural equation modeling: Applications using Mplus2012. null p.

[52] Messman-Moore TL, Long PJ. The role of childhood sexual abuse sequelae in the sexual revictimization of women: An empirical review and theoretical reformulation. Clinical psychology review. 2003;23(4):537–71. 10.1016/s0272-7358(02)00203-9. 12788109

[53] Nduna M, Jewkes RK, Dunkle KL, Shai NPJ, Colman I. Associations between depressive symptoms, sexual behaviour and relationship characteristics: a prospective cohort study of young women and men in the Eastern Cape, South Africa. Journal of the International AIDS Society. 2010;13(1):44. 10.1186/1758-2652-13-44 21078150 PMC2992477

[54] Maniglio R. Child sexual abuse in the etiology of depression: A systematic review of reviews. Depression and Anxiety. 2010;27(7):631–42. 10.1002/da.20687 20336807

[55] World Health Organization. Depression and other common mental disorders: global health estimates. Geneva,Switzerland: World Health Organization.; 2017.

[56] Eaton L, Flisher AJ, Aarø LE. Unsafe sexual behaviour in South African youth. Social science & medicine. 2003;56(1):149–65. 10.1016/s0277-9536(02)00017-5 12435558

[57] Jewkes R, Dunkle K, Nduna M, Shai NJ. Transactional sex and HIV incidence in a cohort of young women in the stepping stones trial. Journal of AIDS and Clinical Research. 2012;3(5). 10.4172/2155-6113.1000158.

[58] Jewkes R, Dunkle K, Nduna M, Levin J, Jama N, Khuzwayo N, et al. Factors associated with HIV sero-status in young rural South African women: connections between intimate partner violence and HIV. International Journal of Epidemiology. 2006;35(6):1461–8. doi: 10.1093/ije/dyl218 17008362

[59] Dunkle KL, Jewkes RK, Brown HC, Gray GE, McIntryre JA, Harlow SD. Gender-based violence, relationship power, and risk of HIV infection in women attending antenatal clinics in South Africa. The lancet. 2004;363(9419):1415–21. 10.1016/s0140-6736(04)16098-4. 15121402

[60] Gibbs A, Jewkes R, Willan S, Washington L. Associations between poverty, mental health and substance use, gender power, and intimate partner violence amongst young (18–30) women and men in urban informal settlements in South Africa: A cross-sectional study and structural equation model. PLoS one. 2018;13(10):e0204956. 10.1371/journal.pone.0204956 30281677 PMC6169941

[61] Gibbs A, Duvvury N, Scriver S. What Works Evidence Review: The relationship between poverty and intimate partner violence. In: What Works to prevent Violence against Women and Girls Global Programme. SAMRC, editor. Pretoria, South Africa 2017.

[62] Machisa MT, Christofides N, Jewkes R. Mental ill-health in structural pathways to women’s experiences of intimate partner violence. PLoS One. 2017;12(4):e0175240. 10.1371/journal.pone.0175240 28384241 PMC5383260

[63] Ramsoomar L, Gibbs A, Machisa M, Chirwa E, Kane J, Jewkes R. What Works Evidence Review: Associations between Alcohol, Poor Mental Health and Intimate Partner Violence In: What Works to prevent Violence against Women and Girls Global Programme. SAMRC, editor. Pretoria, South Africa 2019.

[64] Senn CY, Hollander JA, Gidycz CA. What works? Critical components of effective sexual violence interventions for women on college and university campuses. Sexual Assault Risk Reduction and Resistance: Elsevier; 2018. p. 245–89. 10.1016/b978-0-12-805389-8.00010-4.

[65] Hollander JA. Chapter 10—Empowerment Self-Defense. In: Orchowski LM, Gidycz CA, editors. Sexual Assault Risk Reduction and Resistance. San Diego: Academic Press; 2018. p. 221–44. 10.1016/B978-0-12-805389-8.00011-6.

[66] Gevers A, Dartnall E. The role of mental health in primary prevention of sexual and gender-based violence. Global health action. 2014;7(1):24741. 10.3402/gha.v7.24741 25226417 PMC4165042

[67] Iverson KM, Gradus JL, Resick PA, Suvak MK, Smith KF, Monson CM. Cognitive–behavioral therapy for PTSD and depression symptoms reduces risk for future intimate partner violence among interpersonal trauma survivors. Journal of Consulting and Clinical Psychology. 2011;79(2):193. 10.1037/a0022512 21341889 PMC3071252

[68] Bryant RA, Schafer A, Dawson KS, Anjuri D, Mulili C, Ndogoni L, et al. Effectiveness of a brief behavioural intervention on psychological distress among women with a history of gender-based violence in urban Kenya: A randomised clinical trial. PLoS medicine. 2017;14(8). 10.1371/journal.pmed.1002371 28809935 PMC5557357

[69] Farchione TJ, Bullis JR. Addressing the global burden of mental illness: why transdiagnostic and common elements approaches to evidence-based practice might be our best bet. Cognitive and Behavioral Practice. 2014;21(2):124–6. 10.1016/j.cbpra.2013.12.003.

[70] Gibbs A, Washington L, Abdelatif N, Chirwa E, Willan S, Shai N, et al. Stepping Stones and Creating Futures intervention to prevent intimate partner violence among young people: cluster randomized controlled trial. Journal of Adolescent Health. 2020;66(3):323–35. 10.1016/j.jadohealth.2019.10.004 31784410

[71] Abramsky T, Devries KM, Michau L, Nakuti J, Musuya T, Kyegombe N, et al. The impact of SASA!, a community mobilisation intervention, on women’s experiences of intimate partner violence: secondary findings from a cluster randomised trial in Kampala, Uganda. J Epidemiol Community Health. 2016:jech-2015-206665. 10.1136/jech-2015-206665 26873948 PMC4975800

[72] Jewkes R, Nduna M, Levin J, Jama N, Dunkle K, Puren A, et al. Impact of stepping stones on incidence of HIV and HSV-2 and sexual behaviour in rural South Africa: cluster randomised controlled trial. Bmj. 2008;337:a506. 10.1136/bmj.a506 18687720 PMC2505093

[73] Daigle LE, Fisher BS, Stewart M. The effectiveness of sexual victimization prevention among college students: A summary of “what works”. Victims and Offenders. 2009;4(4):398–404. 10.1080/15564880903227529.

